# Changes in physical activity levels, lesson context, and teacher interaction during physical education in culturally and linguistically diverse Australian schools

**DOI:** 10.1186/1479-5868-9-114

**Published:** 2012-09-18

**Authors:** Dean A Dudley, Anthony D Okely, Philip Pearson, Wayne G Cotton, Peter Caputi

**Affiliations:** 1 School of Human Movement Studies, Faculty of Education, Charles Sturt University, Allen House, Panorama Avenue, Bathurst, New South Wales, Australia; 2 Interdisciplinary Education Research Institute, University of Wollongong, Northfields Avenue, Wollongong, New South Wales, Australia; 3 Faculty of Education, University of Wollongong, Northfields Avenue, Wollongong, New South Wales, Australia; 4 Faculty of Education and Social Work, University of Sydney, Sydney, New South Wales, Australia; 5 School of Psychology, University of Wollongong, Northfields Avenue, Wollongong, New South Wales, Australia

**Keywords:** Direct observation, Cohort study, Feedback, Adolescents, Sport, Pedagogy

## Abstract

**Background:**

Recent data show that only 15% of Australian adolescents participate in adequate amounts of physical activity (PA) and those students from Asian and Middle-Eastern backgrounds in Grades 6–12 are significantly less active than their English-speaking background peers. Schools have recently been recognised as the most widely used and cost-effective setting for promoting PA among youth and one domain within schools where PA can occur regularly for all youth, regardless of cultural background or socio-economic status, is during physical education (PE).

**Methods:**

This study describes changes in physical activity (PA), lesson context and teacher interaction in physical education over the first two years in culturally and linguistically diverse secondary schools. Grade 7 PE classes in six schools were randomly observed using systematic direct observation (n = 81) and then followed up over the same period (n = 51) twelve months later.

**Results:**

There was no significant decline in moderate-to-vigorous physical activity (MVPA) during PE (*MD = −*4.8%; *p =* .777), but a significant decline and medium negative effect in time spent in vigorous physical activity (VPA) (*MD = −*7.9%; *p* = .009) during PE was observed. Significant declines and large negative effects over time in percentage of PE time spent in management (*MD = −*8.8%; *p <* .001) and the number of observations where teachers promoted PA (*MD* = −20.7%; *p* < .001).

**Conclusions:**

The decline of VPA and teacher promotion of PA in culturally and linguistically diverse schools is of concern. Given the declines in VPA and the increases in time spent in game play, further research is needed to ascertain whether PE instruction could be improved by focussing on skill instruction and fitness in a games-based PE instruction model. Further research for increasing teacher promotion of PA during PE is needed.

## Background

Recent data show that only 15% of Australian adolescents participate in adequate amounts of physical activity [1] and those students from Asian cultural backgrounds, and girls from Middle-Eastern backgrounds in Grades 6–12, are significantly less active than their English-speaking background peers [2]. Schools have recently been recognised as the most widely used and cost-effective setting for promoting physical activity (PA) among children and adolescents [3,4] and one domain within schools where PA can occur regularly, regardless of cultural background or socio-economic status, is during school physical education (PE).

In terms of PA participation during PE, no formal guidelines exist in Australia on levels of student PA during PE. However, the US Centres for Disease Control and Prevention [5] recommend that 50% of PE class time should engage students in moderate to vigorous physical activity (MVPA). Australian physical activity guidelines call for 60 minutes of MVPA per day and that this should be accrued through a variety of avenues, including PE [6]. Unfortunately, little is known about the PA levels of Australian students from culturally and linguistically diverse backgrounds during PE and whether they are meeting the recommendation of 50% of class time spent in MVPA. Furthermore, given their lower levels of PA participation within the Australian population, it is worthwhile investigating the contribution PE is making to accumulation of PA in culturally and linguistically diverse communities and whether these levels of PA change as students’ progress through their secondary schooling.

Promoting PA participation is a key aim of PE curricula [5,7]. To achieve this, PE lessons should dedicate sufficient time to skill development and fitness, whilst reducing the amount of time spent managing students [8-10]. Additionally, the social support adolescents receive from their peers, parents and teachers is a key correlate of PA participation [11]. Teachers have the opportunity during their PE classes to provide social support for their students to be physically active in class via the interaction they have with them. However, the extent to which Australian PE teachers promote PA and spend time providing fitness, movement skill and specific game play instruction, and their changes over time, is not known.

In the absence of longitudinal studies, examining changes within control groups in quasi-experimental studies provides the best available evidence of how student activity and lesson context factors prospectively change in PE. In M-SPAN (Middle School Physical Activity and Nutrition) [12] 430 PE lessons were observed at baseline (Grade 6), 711 at 12-month follow-up (Grade 7), and 708 at 24-months (Grade 8) in 12 control schools. In Grade 6, 49% of PE class time was engaged in MVPA and 6% in skill instruction or practice. Both MVPA and skill instruction or practice declined slightly (by around 1%) at 12- and 24-month follow-ups indicating that, in these schools, student activity and lesson context factors did not significantly change over time throughout middle school. Teacher promotion of PA was not reported in this study. No Australian studies have examined student physical activity participation, the time spent in key lesson contexts, or the social support for participation in PE of students from culturally and linguistically diverse backgrounds over time. The purpose of this study was to examine the percentage of class time spent participating in PA, lesson context and teacher interaction during secondary school PE and how these variables changed over time from Grade 7 to Grade 8.

## Methods

### Design

This paper reports on the longitudinal data from the Physical Activity in Linguistically Diverse Communities (PALDC) project. The STROBE (*Strengthening the Reporting of Observational Studies in Epidemiology)* Statement [13] was followed to ensure the transparent reporting of the study.

### Setting and participants

Six secondary schools from south-western Sydney, Australia were identified by the New South Wales Department of Education and Communities (NSW DEC) as having a high proportion of students from culturally and linguistically diverse (CALD) backgrounds. Six schools were invited to participate in the study. Four of the schools were single-sex (two boys and two girls schools) and two were co-educational. In each school, all enrolled Grade 7 (first year of high school in NSW) students were invited to participate. Follow-up data were collected from all six schools in the same half of the school year in the following year when the same students were enrolled in Grade 8.

### Variables

Primary outcome variables for this study were PA levels, lesson instruction context and teacher promotion of PA within PE. As this was a descriptive study, predictors such as age, gender, and school-type were not accounted for. Individual participant demographic data were collected at baseline only and included sex, age, cultural background (based on language most spoken at home) and socioeconomic status (based on postcode of residence).

### Measurement

Physical activity levels, lesson context and teacher promotion were measured using direct observation of randomly scheduled PE lessons (n = 81 baseline, n = 51 follow-up) on separate days at each school over two 6-month periods from July to December, 2008 and from July to December 2009. The System for Observing Fitness Instruction Time (SOFIT) [14] was used to obtain simultaneous recordings of these variables. In brief, the physical activity levels of four randomly selected students, the lesson context, and teacher interactions in relation to promoting PA, were coded every 20 seconds throughout the PE lesson on a rotational basis. The activity codes in SOFIT have been calibrated using heart rate monitoring [14,15] and validated using accelerometers [16]. Observations for a PE lesson began when 51% of students in the class were changed into their PE uniforms and reported to the teaching area and ended when 51% left the teaching area to get changed back into their regular school uniform. This time period was recorded as *useable PE class time*[14]. Students not physically participating in the PE lesson due to injury or illness were not included in the observations [14].

Coding of the activity levels, lesson context, and teacher interaction occurred at the end of each 20-second observation interval. Regarding lesson context, a decision was then made whether class time was being allocated for general content (such as management) or for subject matter (PE) content. If substantive PE content was occurring, an additional decision was made whether the focus was on knowledge content (coded as either general knowledge or physical fitness knowledge) or on motor content (physical activity). If motor content was occurring, a further coding of whether the context was one of fitness, skill practice, game play or free play was made [14]. Movement skill instruction/practice was only coded as such if the motor content being taught was explicitly linked to a movement skill(s). In other words, skills being executed in a game play without explicit instruction from the teacher were game play and not skill practice.

Classifying PA levels was made by observing randomly selected students (one at a time) and determining their level of PA (or active engagement level). The PA/active engagement level provides an approximate intensity of a student's PA. The PA/active engagement levels of 1 to 4 are used describe the body position of the student (lying down, sitting, standing, walking). The highest activity intensity of 5 (very active) describes when the student is expending more energy than they would during normal walking. PA coding was based on the observed activity intensity of the target student at the moment the observation interval ended [14].

Teacher interaction was classified into one of two categories. The first category ‘promotion of physical activity’, related to the teacher verbally or physically encouraging the student to be active. It also included the teacher demonstrating an activity or skill and participating with a student in a way that was perceived as supporting them to be active. The second teacher interaction category was no promotion of physical activity and referred to when none of the previously mentioned teacher behaviours were being observed in the teacher’s interaction with their students [14]. Both the lesson context and teacher interaction categories of SOFIT were derived from definitions commonly used in both PE teacher training and PE pedagogy [17-19].

Four research assistants were trained as SOFIT observers and conducted all the observations. Their training included lectures, video assessment, and live field practice. On completion of the training, the observers were only allowed to commence the observations for this study when an interrater agreement of 85% or more on all variables on pre-recorded “gold-standard” DVDs and during live field practice was reached. On completing each lesson, observers checked all recording forms for missing data. The first author (DAD) entered interval-by-interval codes into a computer for storage and analysis. Accuracy of data entry was maintained by cross checking 100% of raw data points with corresponding cases in the data file until entry was 100% correct. All cases were cross-checked.

### Demographic data

Language mostly spoken at home was collected at baseline on each student from enrolment records. Cultural background was categorised according to region based on the Australian Standard Classification of Languages [20]. Categorisations included in this study were Northern European including English, Southern European, Eastern European, African, Middle-Eastern, Southern-Asian, South-East Asian, East Asian, and Pacific Islands. Enrolment records were also used to ascertain a measure of socioeconomic status based on postcode of residence using the Index of Relative Socio-Economic Disadvantage (IRSD) [21]. To categorise SES, an IRSD score was allocated to each student and then compared with the decile stratifications of the Australian population. Deciles scoring 1 indicated the greatest socio-economic disadvantage and scoring 10 indicated the greatest socio-economic advantage.

### Efforts to minimise bias

Several steps were taken to minimise bias in this study. These steps included recruiting SOFIT observers from pre-service PE teacher courses from two different Australian universities. At no stage were the observers aware of the objectives of the study and observers were required to undertake SOFIT observations at all six schools. This was done to prevent bias of observer observation and variety of interpretations across the schools. Ten field-based interrater reliability checks (8% of the total observations) were conducted during the 12-month observation period. During reliability checks, two observers independently coded the same students in the same PE lesson while being paced by synchronised MP3 players. A percentage of interrater agreement (IRA) was calculated for each variable using the following formula: [#agreements/(#agreements + #disagreements)] × 100. During the assessment period at both baseline and follow-up, ten lessons (8% of the total observations) were observed for reliability with minimum IRAs for student activity levels being 90%, 87% for lesson context, and 96% for teacher behaviour.

Ethics approval was obtained from the University Human Research Ethics Committee and the NSW Department of Education and Communities. Informed consent was provided by the school principals, teachers, students and their parents. Consent was obtained via written consent or if language was not English, New South Wales Department of Education and Communities language staff followed up for parental and student consent via a telephone call to the family’s home.

### Quantitative variables

SOFIT was designed as a lesson-level measure. For basic descriptive statistics, the unit of analysis was a single lesson. For all other analyses, elemental observations made at 20-second intervals were aggregated and lesson context specific summaries were made of each lesson. This yielded up to 18 cases per observed lesson (3 school types × 6 lesson contexts).

The variables reported in this paper included: class factors, student activity levels, lesson context, and teacher interaction. Class factors were lesson length, and school-type. Student activity levels were the proportion of time spent in each of the activity levels. Time spent in MVPA was obtained by summing the proportion of lesson time spent in the “walking” and “very active” categories. Lesson context variables included the proportion of time spent in the six lesson contexts. Teacher interaction variables were the proportion of time spent in two teacher interaction categories (PA promotion and no PA promotion).

### Statistical methods

Percentages were calculated for all SOFIT data (student PA level, lesson context, and teacher interaction) for the entire sample at baseline and follow-up. Unadjusted means, mean differences and effect sizes using Cohen’s *d* were then calculated for PA intensity, lesson length, lesson context, and teacher interaction for baseline and follow-up. In order to be consistent with the reporting of effect sizes in other educational literature, effect sizes were reported as negative *d* values if a reverse effect was observed [22]. School-types, individual schools and classes were not analysed due to lack of statistical power and the variability in the number of classes, teachers, and class size in each of the different schools. All data were analysed using Statistical Package for Social Science (SPSS) version 14.

## Results

### Participants

**Figure 1:**
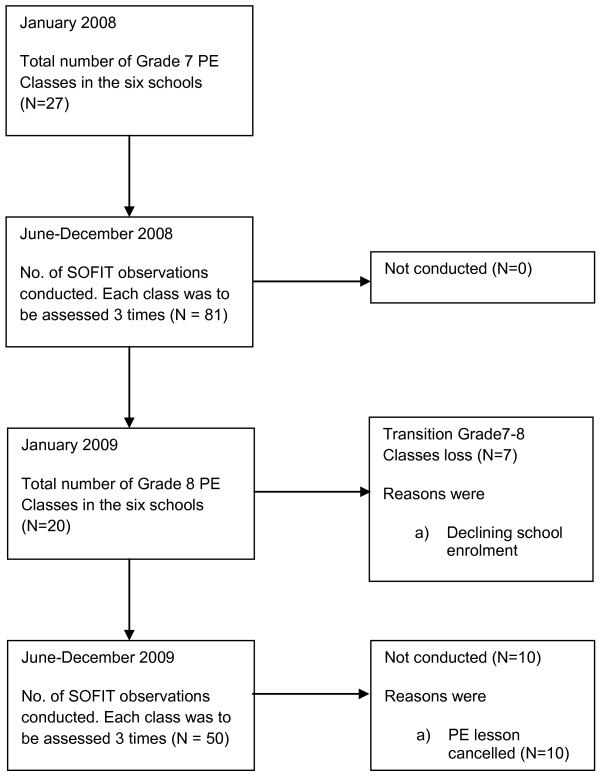
Flowchart for the study.

A flowchart of the study can be reviewed in Figure 1. All teachers at the six schools who taught Grade 7 PE (n = 27) consented to having at least three PE classes being observed. The average class size was 24 students (range 14–27) at baseline. At follow-up, the number of observable PE classes had dropped from 27 to 21 with an average class size of 23 students (range 14–25). This decrease in the number of classes was due to the declining number of student enrolments in these schools which resulted in a reduction in actual number PE classes being taught. Seven classes were observed twice and two classes were only observed once. The reason three observations were not performed on classes were if the observation for that class had been cancelled (for school administration reasons) and catch up observations were not possible before the completion of the school year. There was no active withdrawal from the study by participants or the schools.

A total of 658 Grade 7 students were enrolled in the six schools. Five hundred and eighty six (586) students (Boys, n = 266; Girls, n = 320; 89% of those enrolled) consented to demographic data being collected from school enrolment records at baseline. At follow-up in Grade 8, there were 504 (Boys, n = 225; Girls, n = 279; 77% of those enrolled at baseline) of the consenting students still enrolled in the participating schools.

### Descriptive data

Slightly more participants were female (55%) with the all girls’ schools contributing the largest proportion (40%) at baseline. The mean age of participants at baseline was 12.8 years (*SD* = 0.5). Whilst English was the most common language spoken at home (38%), it was well below the national average of 84% [22]. Middle-Eastern and East Asian dialects were the next most common language spoken at home (36% and 13% respectively) and were well above the national average of 1.2% and 2.3% respectively [22]. Based on postcode of residence, 59% of participants resided in suburbs in the five deciles of greatest socio-economic disadvantage [23]. Demographic data were not collected at follow-up.

## Main results

The percentage of lesson time for student activity levels, lesson contexts, and teacher interactions at baseline and follow-up are reported in Table 1. Walking (36.1% and 39.1%) followed by sitting (31.7% and 25.5%) were the most frequent activity types students engaged in during PE at baseline and follow-up, respectively. At baseline and follow-up, students spent 17.5% and 16.3% of time during PE standing whilst 20.8% and 12.9% of student activity would be considered very active at both collection points, respectively. Most time during PE was spent in MVPA at both baseline and follow-up (56.9% and 52.1%, respectively). There was no statistically significant decline in MVPA from baseline to follow-up (Mean Difference [*MD*] = −4.8; *p* = .777) but a mean loss of 2.7 minutes of MVPA per PE lesson as students progressed from Grade 7 into Grade 8.

**Table 1:** **Unadjusted means, standard deviations, ranges of lesson proportions (%) / mean differences (*MD*), *p *values and Cohen’s *d *values for student activity levels, lesson contexts, and teacher interactions at baseline and follow-up**

** Category**	**Percentage of lesson (n = 81)**			**Percentage of lesson (n = 51)**			**Change from baseline to follow-up**		
	**Baseline (2008)**								
				**Follow-up (2009)**					
	**M**	**SD**	**Range**	**M**	**SD**	**Range**	MD*	p value**	Cohen’s d ***
Student Activity									
Lying down	0.2	0.6	0.0 - 4.4	0.2	1.1	0.0 - 8.0	0.0	*p =* .818	*d =* 0.00
Sitting	25.5	16.4	0.0 – 67.3	31.7	24.1	0.0 - 100	6.2	*p = .*503	*d =* 0.30
Standing	17.5	11.0	1.1 – 45.6	16.3	10.5	0.0 - 43.6	-1.2	*p = .*563	*d =* -0.11
Walking	36.1	14.4	5.6 – 74.5	39.1	18.5	0.0 - 84.4	3.0	*p = .*117	*d =* 0.18
Very active	20.8	11.1	2.1 – 57.1	12.9	12.7	0.0 - 62.9	-7.9	*p =* .009	*d =* -0.66
MVPA^	56.9	18.7	22.2 – 96.3	52.1	24.1	0.0 – 97.9	-4.8	*p =* .777	*d =* -0.22
Lesson Context									
Management	30.8	13.2	3.6 – 67.2	22.3	10.7	0.6 – 49.4	-8.5	*p < .*001	*d =* -0.71
Knowledge	9.5	8.1	0.0 – 44.4	14.2	24.3	0.0 – 96.8	4.7	*p = .*242	*d =* 0.26
Fitness	7.1	11.4	0.0 – 71.7	10.6	18.1	0.0 – 86.3	3.5	*p = .*191	*d =* 0.23
Skill practice	6.2	10.9	0.0 – 44.8	5.2	14.6	0.0 – 75.8	-1.0	*p = .*644	*d = -*0.08
Game play	43.5	24.2	0.0 – 94.6	46.6	28.0	0.0 – 99.4	3.1	*p = .*199	*d =* 0.12
Other (free play)	2.8	5.0	0.0 – 27.2	1.3	3.4	0.0 – 14.6	-1.5	*p = .*408	*d = -*0.35
Teacher Interaction									
Promotion of PA	30.8	19.4	0.0 – 89.4	10.1	8.2	0.0 – 30.3	-20.7	*p < .*001	*d =* -1.39
No promotion of PA	68.9	19.4	10.6 – 100	89.7	8.6	65.6 - 100	20.8	*p < .*001	*d =* 1.39
	Baseline (2008)	Follow-up (2009)							
Mean of usable PE class time^#^ in minutes (Range)	59 (19–110)	59 (29–88)							
Number of lessons with									
>50% MVPA (%)	49 (60.5)	32 (62.7)							
>25% Skills practice (%)	8 (9.9)	4 (7.8)							
>35% Promotion PA (%)	27 (33.3)	0 (0)							

*Mean Difference based on test of within-subjects contrast; **p value represents statistical significance based on test of within-subjects contrast; *** Cohen’s *d* = M *Follow-up–* M *Baseline/* SD *pooled*, ^ MVPA is the sum of the walking and very active categories; ^#^ Usable PE class time defined as when 51% of students arrive in the instruction area to when 51% of students depart instruction area.

Table 1 also shows the *MD*, *p* values and Cohen’s *d* effect size values for physical activity coded variables, lesson context, and teacher interaction over the study period. There was a significant decline and medium negative effect in the percentage of class time spent in the ‘very active’ category (*MD* = −7.9; *p* = .009) which is classified as vigorous physical activity (VPA).

In terms of lesson context, the majority of PE time was spent in ‘game play’ (43.5%) which did not change at 12-month follow-up (*MD* = 3.1; *p* = .199). The percentage of PE class time spent in ‘skill practice’ or explicit skill instruction also remain relatively unchanged from 6.2% to 5.2% (*MD* = −1.0; *p* = .644). This equated to only 2.9 minutes of skill instruction and practice during PE at 12-month follow-up. Regarding teacher interaction and the ‘promotion of student activity’, the time teachers spent encouraging and promoting their students to be physically active dropped significantly from 30.8% to 10.1% of PE class time (*MD* = −20.7; *p* < .001) at 12-month follow-up. Approximately 60% of the lessons at each time point met the CDC recommendation of at least 50% of class time in MVPA. Whilst no formal guidelines exist for skill instruction and practice or teacher promotion of PA, other studies [9,10] have reported proportions of around 30% (25% and 35%, respectively). Only 8 (9.9%) lessons at baseline and 4 (7.8%) at follow-up spent more than 25% of class time engaged in skill instruction and practice. Furthermore, in only 27 (33%) lessons at baseline and 0 (0%) lessons at follow-up did the PE teacher spend more than 35% of the lesson time promoting PA. The average number of useable minutes available for PE instruction over the study period remained constant at 59 minutes.

## Discussion

The purpose of this study was to examine changes from the first to second year of secondary school in PA levels, lesson contexts and teacher interaction among secondary school students from culturally and linguistically diverse backgrounds during compulsory PE in NSW. To the best of our knowledge, this is the first study to observe changes over a 12-month period in these variables in this group of students.

### Changes in physical activity levels during PE

There was a significant decrease and medium negative effect in the percentage of PE class time students spent being ‘very active’ (engaging in vigorous physical activity; VPA) (20.8% to 12.9%; MD = −7.9). The decreases in VPA participation found in this study differ from the findings of another study among similarly aged participants. McKenzie et al. [12] reported that time spent in VPA in the control group of their middle-school intervention stayed relatively consistent from baseline to 12-month follow-up (4.6% to 5.0%; *MD =* 0.4). There are several possible reasons for the significant decline found in the current study. First, this decline in VPA during PE may be reflective of the age-related decline in PA that occurs during adolescence [24]. Second, most of the decline in VPA during PE was explained by the decline occurring in co-educational schools (which had co-educational PE classes). Vigorous physical activity in all girls’ and all boys’ schools declined around 4% and 1%, respectively, whereas the decline in co-educational schools was just under 18%. The reasons for the greater decline in co-educational schools may be that, unlike all girls’ and all boys’ schools, co-educational schools also experienced increases in time spent in management and decreases in time spent in game play. We found in this sample that, at baseline, time spent in management was negatively (*r = −*.218; *p* = .306) and time spent in game play was positively (*r =* .518; p = .010) correlated to MVPA participation in co-educational schools. This suggests that lesson context plays an important role in determining PA participation patterns of students during PE.

### Changes in lesson context during PE

This study found that a substantial proportion of PE lesson time at baseline was spent in management (30.8%, 18.3 minutes) and that this significantly declined at follow-up (22.3%, 13.1 minutes; *p* < .001). These proportions are higher than those found in other studies in Australia (18.8%;) [25], Asia (14%); [26] and the United States of America (9.3%) [12] but the decline is in contrast to the control group in MSPAN study [12] which reported no change from Grade 6 to Grade 8. A possible reason for the greater amount of time spent in management at both time points may be the high proportion of children from non-English speaking backgrounds in the sample, with only 38% of the students speaking English at home. This may suggest that language barriers increase the time teachers need to spend managing students as this context includes aspects such as giving verbal instructions. The significant decline in time spent in management overall was largely driven by the changes in the all girls’ and all boys’ schools (*MD = −*14.6%; -23.4%, respectively). Conversely, co-educational schools experienced a small increase in management time during PE (*MD =* 3.3%). This suggests that these teachers found it easier to manage students in single-sex classes, which is supported by Derry and Phillips [27] in their study of teacher management time in all girls’ versus co-educational PE classes. The decline in management time may also be explained by the increases observed in game play which resulted in concomitant decreases in management time.

The other areas of lesson context that are key to promoting physical activity participation in PE are skill practice and fitness instruction [11-13]. In this study, there was no significant reduction in skill practice (*MD = −*1.0%) during the 12-month study period but skill instruction remained a relatively small teaching component of the lesson. This decline is similar to those found in the M-SPAN study (*MD = −*0.8%) [12] and suggests that PE teachers spend very small proportions of time instructing and practicing skills as student’s progress through secondary schooling. A possible reason for this is that PE teachers see the teaching of movement skills as less important as students’ age. There is evidence to suggest that unless these skills are learned during the early years of schooling, youth are unlikely to achieve high levels of movement competency [28]. It may also be that PE teachers find it difficult to engage students when teaching them skills.

### Changes in teacher interaction during PE

This study also found that teachers spent just under one-third of PE class time (30.8%) promoting physical activity in Grade 7 and that this percentage significantly declined by Grade 8 to 10.1% (*MD* = −20.7%, *p* < .001). None of the lessons observed at follow-up reported more than 35% of class time promoting physical activity. Furthermore, the declines were consistent across all three school types. There have been no known studies reporting on changes in promotion of PA by teachers during PE but a cross-sectional study in the US found that around 35% of class time in middle school was spent promoting PA [29]. Whilst there are no clear justifications for why promotion of PA would drop significantly as students move through secondary school, one explanation could be the autonomy students seek as the move through adolescence and teachers giving them more responsibility for their own physical activity as a result. There is some evidence from longitudinal studies that adolescents’ increase their desire for choice over their own educational experiences, especially as they move through Grades 6 and 7 [30]. This desire for students to have great choice over their learning is controversial within the education discipline though. The effect of student choice over their learning experiences is considered to have a higher effect on motivation outcomes (*d =* .30) [22] than on student learning (*d* = .04) [22]. Furthermore, the more instructionally irrelevant choices had higher outcomes [22] which suggest student choice may not be appropriate in PE but better suited to other aspects of PA promotion in schools. In any case, given that participation in VPA significantly declines over this time, PE teachers still have an important role in promoting physical activity among students as they get older [7], especially in culturally and linguistically diverse communities where PA participation is lower [2].

### Strengths and limitations

A strength of this study was that it was the first known to objectively quantify PA participation, lesson context and teacher interaction at baseline and 12-month follow-up in Australian secondary schools from culturally and linguistically diverse communities. The main limitation of this study was that it reported findings from a small number of schools and that the number of lesson observations declined into the second year. This made it difficult to infer data on the effect of school-type. Another limitation was that data were only collected at two time points which does not allow for trends to be determined. Further longitudinal research is needed to ascertain whether any trends in MVPA, skill instruction and practice time, and teacher promotion of PA exist as students’ progress into their latter years of compulsory secondary physical education. Finally, data were only collected over half an academic year and the subject matter/content of the classes was not recorded. As such, we were unable to determine with these data if annual seasonal issues, such as weather, PE program choices, or subject content influenced the results.

### Implications of findings

This study suggests that NSW PE teachers are delivering CDC recommended levels of MVPA during PE and that their predominant means of doing so is via ‘game play’ during their lessons. More research is needed to ascertain what support NSW PE teachers need instructional model professional development and school structure/policy to keep students physically active whilst students are engaged in ‘game play’ and still teach other outcomes set by the Health and Physical Education syllabus such as movement skills, tactics strategies, teamwork, and problem-solving.

Furthermore, it appears from this study that PE being implemented in NSW secondary schools should examine how it may alleviate the decline in VPA participation and teacher promotion of PA. The declines in promotion of PA are especially cause for alarm as teachers should be expected to maintain adequate levels of feedback and encouragement in order to promote participation in PE.

## Conclusion

Although it appears NSW schools with high proportions of culturally and linguistically diverse students are able to reduce the amount of time they spend managing students during PE, it is the decline in this VPA and teacher promotion of PA that is of concern. Given the declines in VPA and the increases in time spent in game play, further research is needed to ascertain whether PE instruction could be improved by focussing on skill instruction and fitness in a games-based PE instruction model. Finally, further research for increasing teacher promotion of PA during PE is needed and this may need to occur in the form of professional development programs.

## Competing interests

The authors declare that they have no competing interests.

## Authors’ contributions

DAD, ADO, and PP conceptualized and designed the study. DAD and WGC collected the data. DAD and PC conducted the statistical analyses. DAD, ADO, PP, WGC and PC contributed to the writing of the manuscript. All authors read and approved the final manuscript.
